# Commentary: Development and validation of a multivariate predictive model for cancer-related fatigue in esophageal carcinoma: a prospective cohort study integrating biomarkers and psychosocial factors

**DOI:** 10.3389/fonc.2026.1745972

**Published:** 2026-05-25

**Authors:** Sam Sedighi

**Affiliations:** Division of Thoracic and Upper Gastrointestinal Surgery, Department of Surgery, McGill University, Montreal, QC, Canada

**Keywords:** biomarkers, cancer-related fatigue, esophageal carcinoma, predictive model, nomogram, psychosocial factors

## Introduction

Lan et al. (1) developed and internally validated a multivariable model to predict cancer-related fatigue (CRF) in patients undergoing surgery for esophageal carcinoma. The study enrolled a convenience sample from a single tertiary centre in Henan Province, China, assessed fatigue using the Revised Piper Fatigue Scale (RPFS) on postoperative day three, and incorporated psychosocial scales [Hospital Anxiety and Depression Scale (HADS), Pittsburgh Sleep Quality Index (PSQI)] and laboratory biomarkers as candidate predictors within a prospective cohort design. Validation was performed through a 70:30 random split of the same cohort. This topic is clinically relevant, and the effort to integrate biological and psychosocial variables is conceptually sound. However, several design and analytical features may influence the interpretation and generalizability of the reported model performance.

One important concern is that the outcome (RPFS score) and several predictors (HADS anxiety/depression and PSQI sleep quality scores) are patient-reported outcomes (PROMs) assessing overlapping symptom domains. Because these variables are intertwined and collected using similar self-report methods and share conceptual content−fatigue, mood disturbance, and sleep quality are closely interrelated constructs−observed associations may partly reflect shared method variance rather than independent predictive relationships (2, 3). Such overlap could inflate apparent model discrimination and introduce conceptual circularity, whereby predictors effectively capture elements of the outcome construct itself. In this study, the resulting training-cohort AUC of 0.962 may therefore overestimate the model’s true ability to discriminate patients at risk of CRF from those who are not, particularly when applied to populations assessed with different instruments or measurement conditions.

The definition of fatigue also deserves attention. CRF was defined as an RPFS average score ≥1, a threshold that includes minimal postoperative tiredness. Given that fatigue is common−if not expected−on postoperative day three, this cutoff likely captures a broad range of normal recovery experiences. The resulting prevalence of 70.67% suggests that many patients classified as having CRF may have been experiencing transient, mild symptoms. A more stringent threshold, aligned with moderate-to-severe fatigue levels as described in NCCN guidance, might have yielded a more clinically meaningful outcome. This, in turn, could affect event rates, model calibration, and the balance between sensitivity and specificity, ultimately producing a tool better targeted to patients who would benefit from intervention.

Timing is another important issue. Measuring fatigue on postoperative day three primarily reflects acute physiological stress such as surgical inflammation, residual anaesthesia, pain, and disrupted sleep. While these factors are clinically relevant, they differ from the persistent, disproportionate fatigue typically described in survivorship literature on CRF (4, 5). Consequently, the model may be predicting short-term postoperative recovery patterns rather than sustained cancer-related fatigue. Longitudinal assessment at later time points (for example, one week, one month, and three months postoperatively) would help clarify whether the identified predictors remain stable across recovery phases and whether the model retains its discriminative ability beyond the immediate recovery phase.

Additionally, certain laboratory values reported in the study, such as potassium levels in the 400–500 range, appear inconsistent with standard physiological units (normal serum potassium: 3.5–5.0 mmol/L) (6). While this may reflect unit scaling (e.g., values reported as ×0.01 mmol/L) or institutional data-processing conventions, clarification is essential. Mis-specified units or unacknowledged preprocessing transformations could influence coefficient estimation, complicate clinical interpretation of the nomogram, and hinder reproducibility by other investigators (7).

Finally, validation was performed using a random split within the same single-centre cohort. Although this approach provides an internal assessment of model performance, training and validation samples share institutional practices, patient demographics, referral patterns, and measurement conditions. Such internal partitioning may overestimate both discrimination and calibration compared with performance in external or temporally distinct populations. Independent external validation, or at minimum temporal validation using a separate enrolment period, would provide substantially stronger evidence for generalizability and support confidence in clinical implementation. Adherence to the Transparent Reporting of a multivariable prediction model for Individual Prognosis or Diagnosis (TRIPOD) guidelines would further strengthen transparency and reproducibility (8, 9).

In summary, while the study addresses an important clinical question, aspects of predictor–outcome overlap, permissive outcome definition, early postoperative measurement timing, laboratory reporting clarity, and internal-only validation may influence the interpretation of model robustness and transportability. Clarification of the issues identified above and, where feasible, external validation would enhance confidence in the model’s clinical applicability.
